# A Report of Three Surgical Cases, with Comments

**Published:** 1899-10

**Authors:** E. C. Davis

**Affiliations:** Atlanta, Ga.


### A REPORT OF THREE INTERESTING SURGICAL CASES, WITH COMMENTS.

By E. C. DAVIS, A.B., M.D.,
ATLANTA, GA.

The first case which I wish to report shows the lack of reliance which can be reposed in temperature and pulse in attacks of appendicitis; for at no time in this case did the temperature go higher than $100\frac{4}{5}$° F. or pulse above 90; the temperature, on the other hand, was about 99° F.; yet, on operating, the appendix was found ruptured and gangrenous, with distinctive evidences of a beginning active peritonitis.

In the second case, the interest centers especially in the large size of the tumor removed, with the lack of any subsequent unpleasant symptoms. Patient is now apparently well, and regularly employed in her duties as a cook.

CASE 1. February 13, 1899; W. C. P., aged twenty-five, male; occupation, motorman. Was called on the morning of the above date, during the extreme cold, to see patient, who had been suffering great pain in the right side for two days previous, and had taken an opiate for supposed flatulent colic. When I arrived he was still suffering somewhat, but not as severely as prior to the use of the opiate. I found the patient with a temperature of $99\frac{2}{5}$° F., the pulse 80. Great tenderness in the right iliac region, with evidences of a tumor in this locality. The muscles were extremely rigid and great tenderness upon pressure. Diagnosis in this case, appendicitis. Drs. J. B. S. Holmes and J. D. Cromer concurred in diagnosis.

Operation, February 14, 1899. The usual straight incision was made directly upon the tumor, exposing a perforated, gangrenous appendix, with an abscess that seemed walled off from the adjoining cavity by a fold of omentum. This was removed, together with the omental adhesions, carefully packing gauze around the field to prevent an infection of the surrounding area. Enough of the proximal portion of the appendix was found to be healthy to warrant its easy removal, and the incision closed with silk sutures. The incision was closed without drainage, after thoroughly cleansing the area by sponging. Patient made an excellent recovery.

CASE 2. April 29, 1899; M. P., aged thirty-three, colored; occupation, cook. Called in March to see patient, whom I found suffering from circumscribed peritonitis, as the result of an injury to an enormous myo-fibroma of the uterus. The tumor was first noticed by her three years ago, and had increased markedly in size until the present enormous proportions were reached. It was now making pressure on the diaphragm, producing dyspnea. On vaginal examination, a mass could be felt, filling the entire pelvis, irregular in outline and extremely hard, the tumor extending considerably above the umbilicus. At this time patient was kept quietly in bed until all symptoms of peritonitis subsided. This case was also seen by Drs. J. B. S. Holmes and J. D. Cromer, in which the diagnosis was concurred in, and the advice to remove the tumor given.

On April 28th, with the assistance of Dr. Holmes, a myo-hysterectomy was performed, removing the uterus, the right tube, the left tube and the left ovary, but leaving the right ovary. The right ovary was left on account of the dense adhesions, and the amount of shock to which the patient was being subjected on account of the prolonged anesthesia. The appendix was found bound by dense adhesions to the tumor, and was removed. Adhesions were found in almost every direction, but were carefully broken up and tumor removed. It proved to be a multiple myo-fibromata, extending almost from the pelvis to the diaphragm—filling the former cavity almost completely. This patient made an uneventful recovery.

CASE 3. June 3, 1899; H. C., colored, male; occupation, cooper; residence, Chattanooga, Tenn. Called on May 29th to see patient, who gave a history of pleuropneumonia in February last, severe in character, which was followed by an empyema. This was aspirated by the attending physician, a large quantity of pus evacuated, and a rubber drainage-tube inserted. Shortly after this the patient came to Atlanta and passed into the hands of several physicians here, who attended him until I saw him on the above-mentioned date.

When seen by me, the drainage-tube was still *in situ,* almost buried, however, by unhealthy granulations, with a small sloughing area produced by the pressure of the drainage-tube upon the surrounding tissues. It was almost occluded by being abruptly bent. Around the drainage-tube were several openings, which had been formed by small sloughs of the tissue, from which was regularly discharged a most offensive pus.

An effort was made to have him sent to the local hospital, but he was declined admission; so on June 3d, with the assistance of Drs. C. D. Hurt and J. D. Cromer, I resected the eighth and ninth ribs, irrigated the cavity thoroughly, removing a large quantity of extremely fetid pus, and packed the cavity loosely with iodoform gauze. Patient was put to bed in good condition. The gauze was removed the second day, and the cavity was irrigated twice daily for two weeks with normal salt solution. After this time it was only irrigated once daily.

His recovery has been progressive, although he showed distinctive evidences of nephritis, the abdomen becoming distended, the feet considerably swollen, and a considerable quantity of albumen was present in the urine. Under the use of the fluid extract of Canadian hemp the swelling rapidly subsided, and his improvement has been progressive. He has gained about thirty pounds in weight, looks healthy, and is now engaged in light work.

This case shows the importance of a thorough removal of suppurating material from the pleural cavity as early as recognized, and the possibilities of making a mistake in supposing these cases to be always tubercular in origin.

*English-American Building.*

